# Timing-specific effects of single-session M1 anodal tDCS on motor sequence retention in healthy older adults

**DOI:** 10.1016/j.ynirp.2021.100009

**Published:** 2021-04-12

**Authors:** Rohan Puri, Mark R. Hinder, Melanie Krüger, Jeffery J. Summers

**Affiliations:** a Sensorimotor Neuroscience and Ageing Research Group, School of Psychological Sciences, College of Health and Medicine, University of Tasmania, Hobart, Australia; b Sports and Cognition Group, Institute of Sports Science, Leibniz University Hannover, Hannover, Germany; c Research Institute for Sport and Exercise Sciences, Liverpool John Moores University, Liverpool, UK

**Keywords:** transcranial direct current stimulation, Transcranial magnetic stimulation, Motor learning, Aging, Null, Bayes

## Abstract

Anodal transcranial direct current stimulation (tDCS) may assist in counteracting age-related decline in cognitive and motor functions. The current study investigated the potential impact of anodal tDCS, and the timing of its application, in mitigating age-related deficits in motor sequence learning.

Forty-eight healthy older adults received, over the primary motor cortex (M1), tDCS – anodal and sham at least 1 week apart – before, during or after an explicit sequence-learning task with electrophysiological measures of corticospinal excitability (CSE) and short-interval intracortical inhibition (SICI) also obtained.

Bayesian analyses revealed no generalised benefit of anodal tDCS to motor acquisition and immediate retention. Furthermore, there was not enough evidence to support timing-specific stimulation differences on performance during acquisition and immediate retention. However, performance at delayed retention – measured 24 ​h after acquisition – was *worse* in the anodal (13.1%) than sham (17.6%) tDCS session for the group receiving tDCS *during* sequence acquisition, but not before (anodal: 18.4%; sham: 16.7%) or after (anodal: 18.5%; sham: 16.3%) it. No corresponding task-specific stimulation-based changes in CSE and SICI were observed.

Thus, single-session M1 anodal tDCS in healthy older adults not only proved ineffective in facilitating sequence acquisition and immediate retention but also, when administered during sequence learning, proved *detrimental* to delayed retention. Overall, these null and negative results may have implications for the use of tDCS in clinical and rehabilitative settings, especially in the elderly.

## 1 Introduction

Age-related detriments in the motor domain result in coordination difficulties, balance and gait deficits, as well as a generalised slowing of movement (Seidler et al., 2010). These detriments may reflect age-related reorganization of the brain’s functional and structural connectivity patterns (Damoiseaux, 2017) and reduced learning-dependent plasticity (Mary et al., 2015, 2017). As declines in movement control affect the ability of the elderly to carry out everyday activities, learn new skills, and maintain their independence, a variety of approaches are being investigated to enhance neuroplasticity.

One such approach that has shown promise in facilitating motor skill learning, especially in younger adults, is transcranial direct current stimulation (tDCS) (Hsu et al., 2015; Perceval et al., 2016; Tatti et al., 2016), involving the delivery of weak electrical currents applied directly to the scalp. Based on the respective increases and decreases in excitability of corticospinal projections from the primary motor cortex (M1) following tDCS with the anode or cathode over this region of interest, it has been surmised that stimulation influences the resting membrane potential of cortical neurones in a polarity specific manner (Nitsche and Paulus, 2000). Pharmacological studies have suggested that the long-term potentiation-like (LTP-like) after-effects of anodal tDCS are mediated by NMDA (Liebetanz et al., 2002; Nitsche et al., 2003) and GABA (Nitsche et al., 2004) receptor activation. Given that NMDA and GABA receptor activation have also been accorded a role in mediating use-dependent plasticity (Bütefisch et al., 2000), it has been proposed that tDCS may be able to influence motor learning (Ammann et al., 2016; López-Alonso et al., 2015).

In young adults, this premise has been realized to a large extent across a range of motor tasks (Antal et al., 2004; Nitsche et al., 2003; Stagg et al., 2011). However, in older adults, while some improvements in motor function have been reported from single-session M1 anodal tDCS (Hoff et al., 2015; Panouillères et al., 2015; Zimerman et al., 2013), there is yet little evidence of sustained positive effects on motor learning (Kaminski et al., 2017; Raw et al., 2016). Moreover, an important factor that has received little attention but needs to be considered before applying tDCS in rehabilitative settings is the optimal timing of stimulation delivery. That is, tDCS can be applied ‘offline’ (i.e., either before or after the performance of a training task) or ‘online’ (i.e., concurrently during the performance of a training task). Importantly, the time at which stimulation is applied may influence different aspects of motor skill learning. That is, tDCS applied before training may prime the cortical networks involved in skill acquisition. When applied during training, tDCS may facilitate processes involved in skill acquisition. Post training application of tDCS may influence consolidation of skill acquisition. Recently, a meta-analysis suggested that online stimulation to M1 may be more effective than offline stimulation in older adults (Summers et al., 2016). However, tDCS applied immediately after training has also been shown to enhance consolidation of motor skill acquisition in an elderly cohort (Rumpf et al., 2017). Thus, there is clearly a need for further systematic research comparing the three different timings of stimulation within the same task protocol.

The main objective of the present study was to investigate the influence of specifically timed anodal tDCS (i.e., before, during, or after) on motor learning and retention in healthy older adults using a randomized, sham-controlled, doubled-blinded approach. Additionally, transcranial magnetic stimulation (TMS) was used to provide electrophysiological assessments of corticospinal excitability and inhibition prior to and following tDCS, acquisition, and retention. Despite the amplitude of motor evoked potentials (MEPs) elicited by single-pulse TMS being commonly used as a measure of the plastic changes induced in the motor cortex by tDCS, rarely have the behavioural effects and physiological effects of tDCS been studied in parallel. Based on the evidence for an age-related reduction in the efficiency of cortical plasticity mechanisms (Bhandari et al., 2016; Burke and Barnes, 2006; Nitsche et al., 2008), it was expected that older adults may benefit more from online that offline stimulation due to interaction of online tDCS with use-dependent LTP-like mechanisms. To test this, a Bayesian statistical framework was adopted, instead of the frequentist null hypothesis significance testing framework, not only because it forgoes the maligned dichotomous “reject/do-not-reject” decision outcome about the null hypothesis, but also because it allows the quantification of support *in favour* of the null hypothesis (Dienes, 2014; Hoijtink et al., 2019; van Doorn et al., 2020).

## 2 Materials and methods

### 2.1 Participants

Forty-nine healthy older adults were recruited from the local community, with one participant withdrawing from the study prior to its completion, resulting in a total sample size of forty-eight participants. The Mini-Mental State Examination (Dick et al., 1984) was used to screen older participants for cognitive integrity, with all participants scoring within a normal range (score ​≥ ​26; Jacqmin-Gadda et al., 1997). Furthermore, participants were screened for contra-indications to tDCS and TMS via a medical history questionnaire, and were free of any known neuromuscular or neurological dysfunction. Of note, professional typists, musicians, and video game players were not specifically excluded from the current cohort but given that no explicit recruitment calls for members of the aforementioned cohorts were made, we do not expect their proportion in our sample to be over and above that which is observed in a healthy elderly population. Participants provided written informed consent prior to participation and all, except two (left-hand dominant), declared right hand dominance. The study was approved by the Tasmanian Human Research Ethics Committee Network and conducted in accordance with the Declaration of Helsinki.

### 2.2 Experimental procedure

Participants attended two experimental sessions (two components in each session) at the same time of day (±30 ​min), a minimum of one week apart. The two sessions, counterbalanced across participants, differed with regards to (a) tDCS condition – anodal or sham, and (b) explicit task sequence order – ‘original’ or ‘mirrored’ (see Section 2.3, 2.4).

**Fig. 1:**
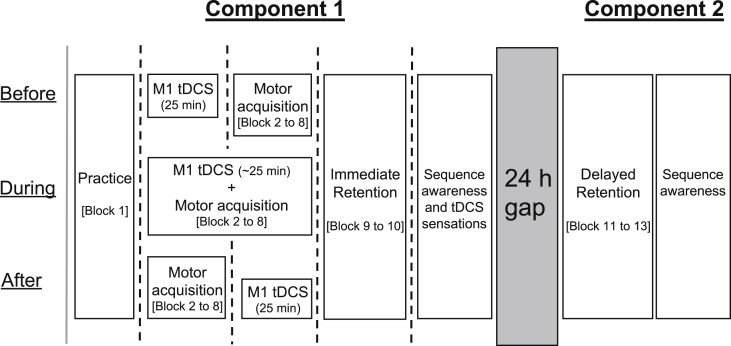
**Experimental procedure.** Forty-eight healthy older adults participated in two sessions (1.5 ​mA anodal and sham M1 tDCS), a minimum of 1 week apart. For each session and all groups, Component 1 began with a baseline TMS block (grey solid vertical line; CSE and SICI) followed by a practice block of the explicit SRTT (Block 1) and another TMS block (black dotted vertical line). Participants received tDCS before, during, or after (*n* ​= ​16 in each group) motor acquisition (Blocks 2 to 8), with participants in the BEFORE and AFTER groups receiving a TMS block in between. Immediate retention (Blocks 9 to 10) was interleaved between two more TMS blocks, with the session ending with the administration of sequence awareness tests and a tDCS sensations questionnaire. Following a 24 ​h retention interval, component two was conducted to probe delayed retention (Block 11 to 13), concluding with sequence awareness tests.

The first component of each session began with motor hotspot and resting motor threshold determination (see Section 2.5), followed by recording of TMS baseline measures. Participants were then given the opportunity to practise a single block of the explicit serial reaction time task (SRTT; Block 1 – ‘practice block’). Following this, participants were randomly assigned to one of three groups which differed according to when tDCS was administered relative to the main explicit SRTT acquisition blocks (Blocks 2 to 8): (a) immediately *before* (*n* ​= ​16; BEFORE group); (b) *during* (*n* ​= ​16; DURING group); or (c) immediately *after* (*n* ​= ​16; AFTER group). Following completion of the acquisition blocks, participants completed two more blocks of the explicit SRTT to investigate immediate retention effects (Blocks 9 and 10). Furthermore, to test for changes in corticospinal excitability (CSE) and short-interval intracortical inhibition (SICI) from the application of tDCS and/or sequence acquisition and immediate retention, TMS was administered at various time points during the experimental procedure (Fig. 1). Participants then completed a tDCS sensations questionnaire and sequence awareness tests (see Supplementary Material 2 and the ‘supplementary_2’ folder at https://osf.io/pk8cv/). For the second component of each session, all participants returned 24 ​h after the start of the first component and completed 3 blocks of the explicit SRTT (no TMS or tDCS administered) to assess delayed retention effects, followed by sequence awareness tests (Fig. 1). Lastly, in light of the importance of sleep on motor sequence consolidation (Diekelmann and Born, 2010), the number of hours slept the night before each component of both sessions was recorded for all participants.

### 2.3 Serial reaction time task (SRTT)

Motor sequence acquisition was assessed by means of the serial reaction time task (Brown et al., 2009; Curran, 1997; Nissen and Bullemer, 1987) and presented in MATLAB 2014a (The MathWorks Inc., Natick, MA) using the Psychophysics Toolbox extensions (Brainard, 1997; Kleiner et al., 2007; Pelli, 1997). Participants sat comfortably at a desk with an LCD monitor ~50 ​cm in front of them, the centre of which was approximately level with their eyes.

Participants were asked to respond as quickly and as accurately as possible to a stimulus (black ‘X’) that appeared at one of four locations (four white squares arranged horizontally across the centre of the monitor against a grey background). Each of the four stimulus locations (termed positions 1–4 from left to right across the screen) required a keypad response with the corresponding finger of the dominant hand assuming a congruent (veridical) spatial mapping between finger position and stimulus location. Thus, for right-handed participants, stimuli position 1, 2, 3 and 4 required responses with the index, middle, ring, and little finger, respectively (reversed finger order for left-handed participants). Following a correct response, the presentation of the next stimulus occurred at a randomized interval of 250–750 ​ms. In the event of an incorrect response, the stimulus remained on the screen until the correct button was pressed.

Stimulus presentation followed a repeating 12-element sequence (‘original’: 1-2-4-3-1-4-2-3-4-1-3-2, or ‘mirrored’: 2-3-1-4-3-2-4-1-3-4-2-1) with an equal number of stimuli presented for each finger (three) and no stimuli presented consecutively (e.g., 1-1). Each block of the SRTT consisted of 10 repeats of the 12-element sequence (i.e., 120 trials) and, following Tunovic et al. (2014), participants were made aware of the presence of a repeated sequence as well as being informed that a blue coloured stimulus (as opposed to black) would indicate the *beginning* of the repeated sequence (i.e., position 1 for the ‘original’ sequence; position 2 for the ‘mirrored’ sequence), but neither the actual sequence nor its length were disclosed. In addition to the 120 sequence trials, 24 random trials were presented at the start and at the end of each block, resulting in a total of 168 trials per block. These random trials enabled us to discriminate between sequence-specific learning and more generalised quickening of motor responses. Accordingly, the task employed represents an explicit sequence-learning task (explicit SRTT) in which we observed both clear age-related performance deficits as well as clear age-related free recall deficits in our pilot study (see Supplementary Material 1 and the ‘supplementary_1’ folder at https://osf.io/pk8cv/). Each participant was exposed to both sequences (original and mirrored) across both sessions of the experiment (e.g., a participant receiving the mirrored sequence in the first session, received the original sequence in the second session, or vice versa), with sequence presentation counterbalanced across participants. Lastly, sequence awareness tests were conducted to probe both free recall and recognition of the sequence (see Supplementary Material 2 and the ‘supplementary_2’ folder at https://osf.io/pk8cv/).

### 2.4 Transcranial direct current stimulation (tDCS)

Direct current stimulation was administered using a battery-operated constant direct current stimulator (HDCStim™, Newronika s.r.l., Milan, Italy) and delivered via two conductive rubber electrodes placed in saline soaked sponges. The anode electrode (5 ​cm ​× ​5 ​cm) was placed over the first dorsal interosseous (FDI) representation of the dominant primary motor cortex (left M1 for right-handed participants; right M1 for left-handed participants) with the cathode electrode (8 ​cm ​× ​6.5 ​cm) placed over the contralateral supraorbital region.

For the anodal condition, participants in the BEFORE and AFTER groups received 25 ​min of 1.5 ​mA anodal tDCS before or after the motor acquisition blocks respectively, with participants in the DURING group receiving 1.5 ​mA anodal tDCS for the duration of the acquisition blocks (see Section 2.2, Fig. 1). Our rationale for a) a 1.5 ​mA tDCS intensity was that it was used in previous work in our laboratory demonstrating significant changes in M1 CSE following anodal tDCS in older adults (Puri et al., 2016), and b) the 25 ​min stimulation duration was chosen as previous research has shown a *reversal* of excitability-inducing M1 anodal tDCS effects at 26, and greater, minutes of stimulation (Hassanzahraee et al., 2020; Monte-Silva et al., 2013), an outcome we did not wish to eventuate. Participants in the BEFORE and AFTER groups were instructed to keep their eyes open and hands as still as possible for the duration of the tDCS protocol. Current was ramped up, over 7 ​s, from 0 to 1.5 ​mA where it was maintained for the duration of the stimulation. For the sham condition, exactly the same procedures were employed as for anodal stimulation, except that the current was stepped down after the initial ramp up period. Both tDCS conditions - anodal and sham - were counterbalanced for each of the three groups and delivered in a double-blinded manner, i.e., the experimenter administering tDCS was not involved in setting up anodal and sham protocols for each participant on the tDCS device. Lastly, to investigate tDCS comfort, a tDCS sensations questionnaire (Fertonani et al., 2010, 2015) was administered at the end of each tDCS session.

### 2.5 Transcranial magnetic stimulation (TMS) and electromyography (EMG)

Surface EMG electrodes (Ag/AgCl) were arranged in a belly-tendon montage to measure EMG activity from the dominant FDI. Signals were sampled (4000 ​Hz), band-pass filtered (20–1000 ​Hz with a 50 ​Hz notch filter), and amplified (gain of 1000) using a 16-bit AD system (CED Power 1401 and CED 1902; Cambridge, UK) to be stored for offline analyses.

A figure-of-eight coil (internal diameter of 70 ​mm), connected to a Magstim BiStim^2^ stimulator (Magstim Company, Dyfed, UK), was used for all TMS procedures. The motor ‘hotspot’ – defined as the optimal scalp location over the contralateral primary motor cortex (left M1 for right-handed participants; right M1 for left-handed participants) where suprathreshold TMS evoked the largest and most consistent MEP in the relaxed FDI muscle – was located and then marked using a felt-tip pen. The TMS coil was held tangentially to the scalp, with the handle pointing ~45° backwards, to ensure that current flow in the brain was in the optimal posterior-anterior direction. Following this, resting motor threshold (rMT) – defined as the minimum intensity (represented as a % of maximum stimulator output) required to evoke motor evoked potentials (MEPs) of ≥50 ​μV in 3 out of 5 consecutive trials (Carroll et al., 2001; Hinder et al., 2010) – was determined for each participant. Single and paired-pulse paradigms were used to assess CSE and SICI (Kujirai et al., 1993). Single-pulse TMS trials involved a single ‘test’ stimulus at 130% rMT, whereas paired-pulse TMS trials involved a conditioning stimulus (70% rMT) 2.5 ​ms before the test stimulus (130% rMT). Each TMS block consisted of 30 TMS trials, with 15 single- and paired-pulse TMS trials delivered in a random order, with the number of TMS blocks administered varying between groups (see Section 2.2). Using visual feedback, participants’ online EMG activity was monitored by the experimenter to ensure the muscles were fully relaxed and when necessary participants were reminded to keep their hand quiescent.

### 2.6 Data processing

Reaction time (RT) for each button press (for both the random and sequence button presses) was calculated as the time taken for a correct response to be registered following stimulus onset (key hit). For each key hit within a block, excessively short or long reaction times were capped at a value lesser/greater than 3 standard deviations of the median RT. This procedure was conducted separately for random and sequence trials within each block and reduced the skewing effect of outliers. Following this, the mean RT for random trials was computed using the 12 trials immediately preceding and following the sequence trials, and the mean RT for sequence trials computed using the 120 sequence trials. Note, our rationale for only using the 12 random trials prior to and following the sequence (instead of all random trials) was to select trials that were most reflective of ‘true’ task behaviour. Specifically, participants may take a few trials to become focussed at the commencement of each block, and towards the end of the block participants may experience fatigue, both of which are likely to affect task-specific performance in ways that are not of interest to the current study. In addition, regarding the averaging of all the sequence repeats in a block, given that the main focus of the current study was to investigate the influence of tDCS, and not temporal aspects of learning, we did not have any plausible rationale as to why tDCS would consistently, across blocks, influence certain sequence repeats over others. Instead, given the timeframe over which tDCS effects are observed, we argue for a stronger rationale to observe differences *between* blocks and hence the sequence trials within a block were averaged. Sequence-specific learning was then calculated by subtracting random and sequence mean RTs, divided by the mean random RT [(mean random RT – mean sequence RT)/mean random RT], expressed as a percentage (Brown et al., 2009). Therefore, higher values indicate greater sequence-specific improvement. Lastly, error rates were not analysed as a measure of motor skill as error rates are known to be extremely low for this task (in the <2–4% range as per Tunovic et al., 2014). Indeed, error rates were in the extremely low ranges for both anodal (random: 3.68%; sequence: 2.25%) and sham (random: 4.10%; sequence: 2.35%) tDCS conditions (see ‘errors.csv’ for raw data on https://osf.io/pk8cv/). Given the extremely low occurrence of these errors, the *practical* significance of a manipulation where the observed error rate differences are in the 1–2% range is likely to be inconsequential, and thus no inferential statistics were conducted.

For neurophysiological measures, peak-to-peak MEP amplitude in the dominant FDI in a time window 10–100 ​ms following TMS was utilized. Trials contaminated with muscle activity – defined as root mean square EMG activity exceeding 0.025 ​mV in a 50 ​ms time window immediately prior to TMS – were excluded from analyses. Average peak-to-peak MEP amplitude (in mV) was then determined for single- and paired-pulse trials separately at every time-point (Fig. 1). Corticospinal excitability was inferred from the averaged single-pulse MEPs and short intracortical inhibition from the ratio of the averaged paired-pulse MEPs to averaged single-pulse MEPs (at each time point). For each group, CSE and SICI measures at all later time-points (black dotted vertical lines, Fig. 1) were normalised to baseline measures (grey solid vertical line, Fig. 1) to control for session-to-session variability. Natural-log transformations were then conducted to address violations of normality and positive skewness associated with normalised data (as undertaken previously in Hinder et al., 2014; Puri et al., 2015, 2016).

### 2.7 Statistical analyses

For behavioural measures (i.e., explicit SRTT performance), three-way Bayesian ANOVAs were conducted to directly compare the stimulation-specific effects of different tDCS timings relative to the SRTT. Specifically, three separate three-way Bayesian ANOVAs were conducted to assess *acquisition* (STIM: Anodal, Sham; BLOCK: 2 to 8; GROUP: Before, During), *immediate retention* (STIM: Anodal, Sham; BLOCK: 9 to 10; GROUP: Before, During, After), and *delayed retention* (STIM: Anodal, Sham; BLOCK: 11 to 13; GROUP: Before, During, After). Note that for the *acquisition* analysis, only the DURING and BEFORE groups were directly compared considering that different stimulations (active anodal vs. sham) were only administered after the acquisition phase for the AFTER group. Lastly, to probe changes in off-line performance over the 24 ​h period, an additional three-way Bayesian ANOVA (STIM: Anodal, Sham; RETENTION: Immediate, Delayed; GROUP: Before, During, After) was conducted comparing the average performance at immediate retention (Blocks 9 to 10) to the average performance at delayed retention (Blocks 11 to 13).

For neurophysiological measures (natural log-transformed normalised CSE and SICI), stimulation dependent effects (active anodal stimulation vs. sham stimulation) were only analysed for each timing group separately (BEFORE, DURING, and AFTER), and not directly, as the number of TMS measurement time-points varied between the three groups (see Fig. 1). Specifically, for the BEFORE and AFTER groups, a two-way Bayesian ANOVA was conducted with factors of STIM (Anodal, Sham) and TIME (4 time-points), whereas for the DURING group, a two-way Bayesian ANOVA was conducted with factors of STIM (Anodal, Sham) and TIME (3 time-points).

For multi-factor ANOVAs, such as those described above, not only do the number of possible models become very large but selecting a ‘best’ model does not take into account model uncertainty. To account for model uncertainty, *all* models were considered and a Bayesian Model Averaging approach was utilized (Hinne et al., 2020) whereby more weight is given to those models that predict the data relatively well and vice versa. Based on these model-averaged results, hypotheses testing for main and interaction effects were conducted by means of Bayes factors (van Doorn et al., 2020). Bayes factor quantifies the relative predictive performance of two competing hypotheses and, in the current study, Inclusion (*BF*_*incl*_) *or* Exclusion (*BF*_*excl*_) Bayes factors for matched models are reported for the main and interaction effects (van den Bergh et al., 2019); for consistency we report the *BF* (*incl* or *excl*) that is greater than 1, with the other *BF* (*excl* or *incl*) reciprocally related to the reported value. Specifically, a *BF*_*incl*_ of value, ‘x’, can be interpreted as “the data are ‘x’ times more likely under models that *include* the effect (main or interaction) than under models without the effect” and a *BF*_*excl*_ of value, ‘y’, as “the data are ‘y’ times more likely under models that *exclude* the effect (main or interaction) than under models with the effect”. Thus, larger values of *BF*_*incl*_ or *BF*_*excl*_ indicate more support for the inclusion or exclusion of an effect, respectively. Though these values of Bayes factors are interpretable in itself, as they are continuous measures of relative evidence, Bayes factors have also been categorised (1–3: “anecdotal” evidence; 3–10: “moderate” evidence; 10–30: “strong” evidence; 30–100: “very strong” evidence; >100: “extreme” evidence) as per Lee and Wagenmakers (2013). In addition, given the known sensitivity of Bayes factors to the specification of prior distributions (Etz et al., 2018; Kruschke and Liddell, 2018), every analysis was conducted with the default “medium” (multivariate Cauchy priors with *r* ​= ​0.5 for fixed effects) and “ultrawide” (multivariate Cauchy priors with *r* ​= ​1 for fixed effects) prior specifications (Rouder et al., 2012). Thus, along with the aforementioned subscript (to indicate Inclusion or Exclusion Bayes factors), Bayes factors are annotated as “*BF_med*” (Bayes factor with “medium” prior specification) or “*BF_uw*” (Bayes factor with “ultrawide” prior specification). Lastly, for every ANOVA, the number of samples was increased until each model had a Bayes factor computation error less than 5% (e.g., a Bayes factor of 20 can fluctuate between 19 and 21 ​at most).

The use of Bayesian, compared to frequentist, statistics in the current study was a considered choice given that no *a priori* power analyses were conducted. Specifically, in a frequentist null hypothesis significant testing framework, a non-significant *p* value (i.e., usually, *p* ​>0.05) may either indicate that the manipulation had no true effect (i.e., evidence of a null finding: “evidence of absence”) or that the sample size was unable to detect a true non-zero effect of the manipulation (i.e., insufficient power: “absence of evidence”), with limited options to disentangle these two alternatives (Keysers et al., 2020). In contrast, the continuous nature of the Bayes factor measure can be interpreted as 1) providing enough evidence to accept the alternative hypothesis; 2) providing enough evidence to accept the null hypothesis (“evidence of absence”); or 3) stating the inconclusiveness of the evidence towards either hypothesis (“absence of evidence”). This property of Bayes factor (i.e., differentiating between “evidence of absence” and “absence of evidence”) as well as the categorisations utilized (i.e., Bayes factors ​> ​3 to make substantive inferences as per Lee and Wagenmakers (2013)) are crucial in reporting null results.

Descriptive statistics are reported as means and 95% credible intervals (95% CIs, reported in square brackets, reflect a 95% probability of the estimate lying within the lower and upper bounds, given the data), unless specified otherwise, and are used in all figures. All analyses were conducted using default settings (unless specified above) in JASP v0.14 (JASP Team, 2020), which uses the ‘BayesFactor’ (Morey and Rouder, 2020) and ‘BAS’ (Clyde, 2020) R packages for ANOVA models and model averaging, respectively. All analyses and datasets (main article and supplementary materials) can be accessed via https://osf.io/pk8cv/.

## 3 Results

All participants in the BEFORE (*n* ​= ​16, mean age ​± ​SD ​= ​65.6 years ​± 3.2, 11 women and 5 men, all right-hand dominant), DURING (*n* ​= ​16, mean age ​± ​SD ​= ​64.4 ​± ​3.9 years, 6 women and 10 men, all right-hand dominant), and AFTER (*n* ​= ​16, mean age ​± ​SD ​= ​66.9 ​± ​6.0 years, 9 women and 7 men, all, but two, right-hand dominant) groups completed both sessions without any adverse effects. Comparing the three groups’ age, the data provided anecdotal to moderate evidence for excluding GROUP as a predictor (*BF_med*_*excl*_ ​= ​2.67; *BF_uw*_*excl*_ ​= ​6.31). In addition, for the number of hours slept before each session, the data provided anecdotal to strong evidence for the exclusion of main and interaction effects involving GROUP (*BF_med*_*excl*_ ​= ​1.50–5.38; *BF_uw*_*excl*_ ​= ​2.89–15.33). Thus, both these results suggest that age and the numbers of hours slept before each session *do not* differ substantially between the three tDCS timing groups.

### 3.1 Explicit SRTT performance

Comparing practice block SRTT performance (Block 1) between the three tDCS timing groups, the data provided anecdotal to strong evidence for the exclusion of main and interactions effects involving GROUP (*BF_med*_*excl*_ ​= ​1.83–5.79; *BF_uw*_*excl*_ ​= ​3.97–16.91), suggesting similar practice block performance.

**Fig. 2:**
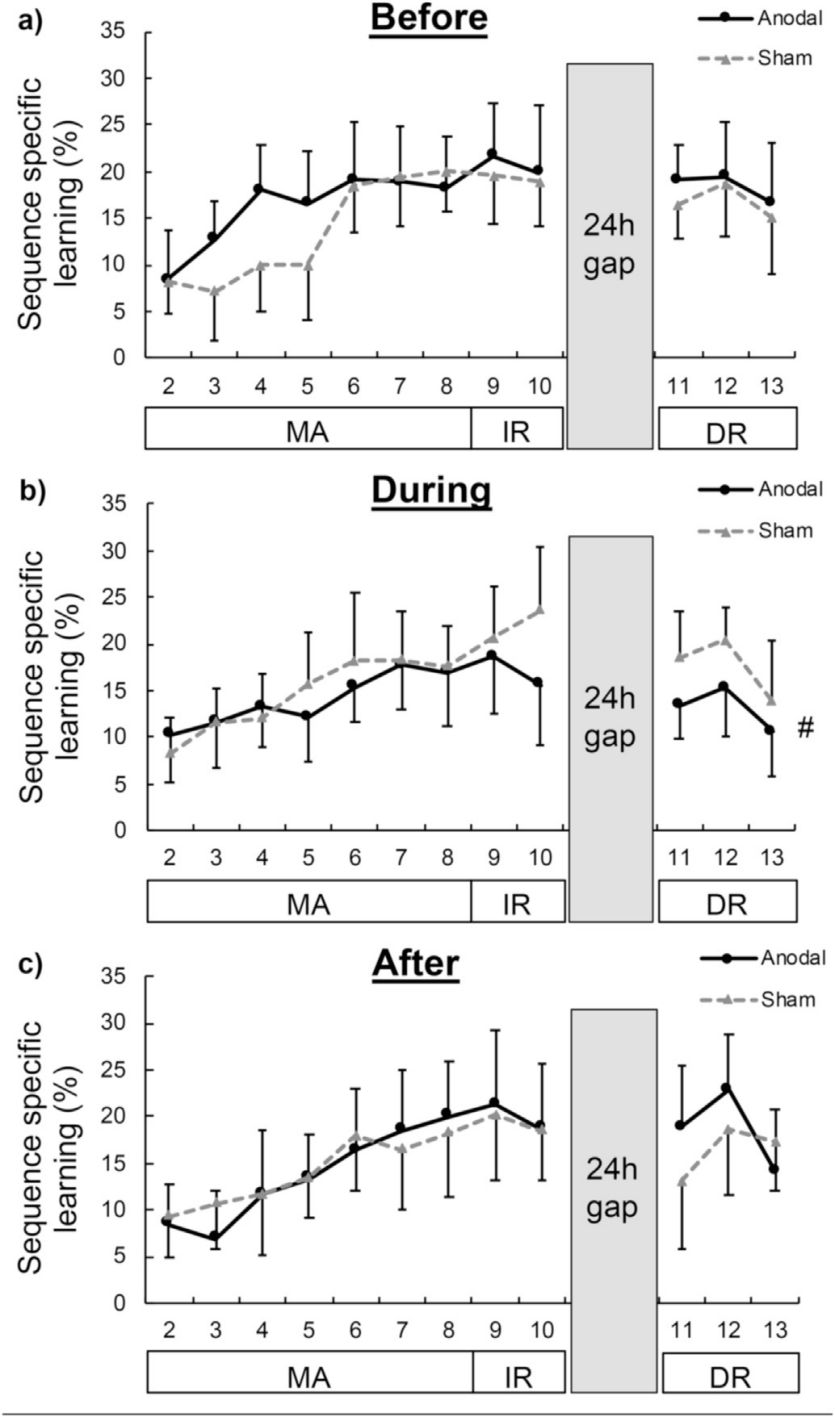
**Behavioural outcomes.** Sequence-specific learning, relative to random key presses, (expressed as a % plotted on the ordinate) during motor acquisition (MA; Block 2 to 8), immediate retention (IR; Block 9 to 10), and delayed retention (DR; Block 11 to 13) plotted separately for anodal (solid black line) and sham (dotted grey line) sessions on the abscissa for the a) BEFORE, b) DURING, and c) AFTER groups (*n* ​= ​16 in each group). The hash symbol (#) indicates worse DR sequence-specific learning for the DURING tDCS timing group, compared to the BEFORE and AFTER tDCS timing groups. Error bars indicate unidirectional 95% CIs around the mean.

Substantial sequence-specific performance improvements were observed for all groups during the motor acquisition phase (Blocks 2 to 8), depicted in Fig. 2, as suggested by the extreme evidence for the inclusion of the BLOCK main effect (*BF_med*_*incl*_ ​= ​6.5e6 – 4.3e13; *BF_uw*_*incl*_ ​= ​3.9e6 – 2.2e13). However, on average, motor acquisition did not vary between stimulation types, as suggested by the moderate evidence for the exclusion of the STIM main effect (*BF_med*_*excl*_ ​= ​3.75; *BF_uw*_*excl*_ ​= ​7.20). Comparing stimulation-dependent differences of the DURING and BEFORE groups, the data provided anecdotal evidence for the inclusion and exclusion of the GROUP ∗ STIM two-way interaction effect (*BF_med*_*incl*_ ​= ​1.57; *BF_uw*_*excl*_ ​= ​1.13) and moderate to strong evidence for the exclusion of the GROUP ∗ STIM ∗ BLOCK three-way interaction effect (*BF_med*_*excl*_ ​= ​4.62; *BF_uw*_*excl*_ ​= ​27.81). As expected, for the AFTER group the data provided moderate to extreme evidence for the exclusion of the main and interaction effect involving STIM (*BF_med*_*excl*_ ​= ​6.52–19.27; *BF_uw*_*excl*_ ​= ​12.68–194.43) as stimulation did not occur until *after* the acquisition phase.

Immediate retention (Blocks 9 to 10) did not vary between stimulation types, as suggested by the moderate evidence for the exclusion of the STIM main effect (*BF_med*_*excl*_ ​= ​4.68; *BF_uw*_*excl*_ ​= ​9.02). Comparing the stimulation-dependent differences between the three groups, the data provided anecdotal evidence for the inclusion and exclusion of the GROUP ∗ STIM two-way interaction effect (*BF_med*_*incl*_ ​= ​1.35; *BF_uw*_*excl*_ ​= ​1.63) and moderate to strong evidence for the exclusion of the GROUP ∗ STIM ∗ BLOCK three-way interaction effect (*BF_med*_*excl*_ ​= ​4.42; *BF_uw*_*excl*_ ​= ​10.99).

Delayed retention (Blocks 11 to 13, conducted 24h following motor acquisition) did not vary between stimulation types, as supported by the moderate to strong evidence for exclusion of the STIM main effect (*BF_med*_*excl*_ ​= ​7.63; *BF_uw*_*excl*_ ​= ​14.87). However, there was moderate evidence for the inclusion of the STIM ∗ GROUP interaction effect (*BF_med*_*incl*_ ​= ​9.93; *BF_uw*_*incl*_ ​= ​5.03). As can be seen in Fig. 2, there were negligible stimulation-dependent differences in performance for the BEFORE (anodal: 18.39% [13.43–23.35]; sham: 16.71% [12.41–21.00]) and AFTER (anodal: 18.54% [12.62–24.45]; sham: 16.30% [10.63–21.96]) groups, but considerably worse delayed retention performance for the DURING group after anodal (13.08% [9.17–16.99]) than sham (17.58% [13.05–22.11]) stimulation. This detriment did not vary across the different blocks as the data provided moderate to strong evidence for the exclusion of the STIM ∗ GROUP ∗ BLOCK interaction effect (*BF_med*_*excl*_ ​= ​3.50; *BF_uw*_*excl*_ ​= ​14.99).

Small decrements in performance over the 24 ​h retention interval was observed as suggested by the moderate to strong evidence for inclusion of the main effect of RETENTION (*BF_med*_*incl*_ ​= ​14.09; *BF_uw*_*incl*_ ​= ​8.56). However, no stimulation-dependent differences in performance over the 24 ​h period were observed considering the moderate evidence for the exclusion of the STIM ∗ RETENTION interaction effect (*BF_med*_*excl*_ ​= ​4.26; *BF_uw*_*excl*_ ​= ​8.38). In addition, no stimulation-dependent group differences in performance over the 24 ​h period were observed based on the moderate to strong evidence for the exclusion of the STIM ∗ RETENTION ∗ GROUP interaction effect (*BF_med*_*excl*_ ​= ​6.38; *BF_uw*_*excl*_ ​= ​17.53).

In summary, M1 anodal tDCS did not improve motor acquisition or immediate retention in healthy older adults, compared to sham tDCS. In contrast, delayed retention was *worse* after the anodal, compared to sham, tDCS session but only when administered *during* motor acquisition and not before or after it.

### 3.2 Neurophysiological measures

Three participants’ neurophysiological data could not be collected due to either high resting motor thresholds (one individual in each of the BEFORE and DURING groups) or technical difficulties in one of the two experimental sessions (one individual in the DURING group). All data are presented as means ​± ​95% CIs unless stated otherwise.

Resting motor threshold did not vary considerably between the anodal (BEFORE: 40.20% [35.71–44.69]; DURING: 39.14% [35.11–43.18]; AFTER: 40.25% [36.15–44.35]) and sham (BEFORE: 40.67% [35.96–45.38]; DURING: 39.36% [35.38–43.34]; AFTER: 39.94% [35.79–44.08]) sessions among the three groups, as suggested by the anecdotal to moderate evidence for the exclusion of the STIM ∗ GROUP interaction (*BF_med*_*excl*_ ​= ​1.63; *BF_uw*_*excl*_ ​= ​3.41).

#### 3.2.1 Corticospinal excitability

Baseline CSE between the BEFORE (anodal: 0.82 ​mV [0.46–1.18]; sham: 0.63 ​mV [0.43–0.83]), DURING (anodal: 0.90 ​mV [0.63–1.18]; sham: 1.01 ​mV [0.56–1.46]), and AFTER (anodal: 0.78 ​mV [0.47–1.08]; sham: 0.79 ​mV [0.51–1.08]) groups differed negligibly depending on the type of stimulation (anodal vs. sham), based on the moderate evidence for the exclusion of the STIM ∗ GROUP interaction (*BF_med*_*excl*_ ​= ​3.02; *BF_uw*_*excl*_ ​= ​7.25).

**Fig. 3:**
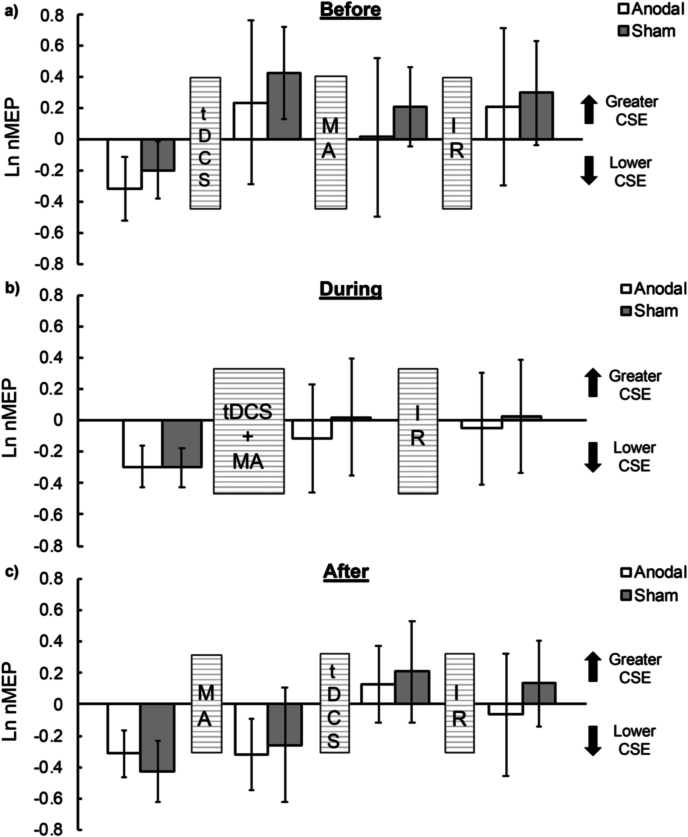
**Corticospinal excitability.** CSE (ordinate; natural log transformed normalised to baseline CSE) at various time-points (abscissa) before and after acquisition (‘MA’), tDCS (‘tDCS’), and immediate retention (’IR’) for the a) BEFORE, b) DURING, and c) AFTER groups following anodal (unfilled bars) and sham (grey-filled bars) tDCS. Values greater than 0 indicate greater CSE relative to baseline CSE and values lesser than 0 indicate lower CSE relative to baseline CSE. Error bars indicate 95% CIs around the mean.

Across the three tDCS timing groups, the data provided anecdotal to very strong evidence for the exclusion of main and interaction effects involving STIM (*BF_med*_*excl*_ ​= ​2.02–10.81; *BF_uw*_*excl*_ ​= ​3.55–45.86), suggesting no stimulation specific effects of anodal tDCS on corticospinal excitability compared to sham tDCS (Fig. 3).

#### 3.2.2 Short-interval intracortical inhibition

Mean baseline SICI values for the BEFORE (anodal: 0.47 [0.28–0.67]; sham: 0.82 [0.42–1.21]), DURING (anodal: 0.54 [0.37–0.71]; sham: 0.47 [0.32–0.61]), and AFTER (anodal: 0.48 [0.32–0.64]; sham: 0.54 [0.34–0.75]) groups were all below 1, indicating that the TMS stimulation parameters adequately captured intracortical inhibitory processes in the primary motor cortex. Despite moderate evidence for the inclusion of a STIM ∗ GROUP interaction (*BF_med*_*incl*_ ​= ​4.79; *BF_uw*_*incl*_ ​= ​3.74) revealing greater inhibition for the BEFORE group in the anodal session compared to the sham session, both mean values were below 1. This baseline difference between the stimulation sessions was attenuated by the normalization procedures employed prior to conducting further analyses (see Section 2.6).

**Fig. 4:**
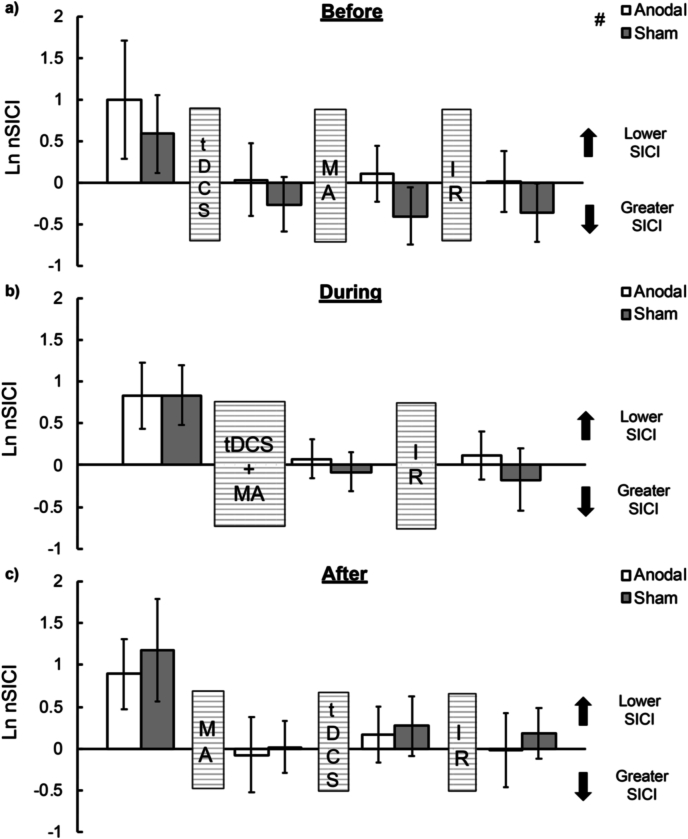
**Short-interval intracortical inhibition.** SICI (ordinate; natural log transformed normalised to baseline SICI) at various time-points (abscissa) before and after motor acquisition (‘MA’), tDCS (‘tDCS’), and immediate retention (’IR’) for the a) BEFORE, b) DURING, and c) AFTER groups following anodal (unfilled bars) and sham (grey-filled bars) tDCS. Values greater than 0 indicate lower SICI relative to baseline SICI and values lesser than 0 indicate greater SICI relative to baseline SICI. The hash symbol (#) indicates lower SICI during anodal, compared to sham, tDCS for the BEFORE tDCS timing group. Error bars indicate 95% CIs around the mean.

For the DURING and AFTER tDCS groups, the data provided anecdotal to very strong evidence for the exclusion of main and interaction effects involving STIM (*BF_med*_*excl*_ ​= ​1.92–10.30; *BF_uw*_*excl*_ ​= ​3.30–45.79), suggesting no stimulation specific effects of anodal tDCS on SICI compared to sham tDCS (Fig. 4). However, for the BEFORE group, greater release of SICI was observed in the anodal (log transformed: 0.287 [-0.07 – 0.64]; raw: 1.33 [0.93–1.90]) than sham (log transformed: 0.115 [-0.362 – 0.131]; raw: 0.89 [0.70–1.14]) tDCS session as suggested by the moderate evidence for the inclusion of the main effect of STIM (*BF_med*_*incl*_ ​= ​9.11; *BF_uw*_*incl*_ ​= ​6.43). This was not modulated across the various TMS time-points as the data provided moderate to very strong evidence for the exclusion of the STIM ∗ TIME interaction (*BF_med*_*excl*_ ​= ​9.95; *BF_uw*_*excl*_ ​= ​43.28).

In summary, anodal tDCS did not enhance M1 CSE in older adults compared to sham tDCS. Anodal tDCS did, however, lead to a generalised release of SICI, compared to sham tDCS, but only for the group receiving tDCS *before* the motor acquisition phase.

## 4 Discussion

The current study aimed to provide, in a group of healthy older adults, insights concerning the potential impact of M1 anodal tDCS, and the timing of its application (relative to an associated motor sequencing-learning task), upon task-specific learning. Based on a randomized, sham-controlled, double-blinded experimental design, three groups of healthy older adults received M1 anodal tDCS either before, during, or after undertaking a serial reaction time task. Our data and Bayesian analyses suggested i) enough evidence to conclude no generalised benefit of M1 anodal tDCS to motor acquisition and immediate retention, ii) not enough evidence to conclude about tDCS timing-based stimulation differences during motor acquisition and immediate retention, iii) enough evidence to conclude that anodal tDCS applied *during* motor acquisition resulted in detrimental performance at delayed retention (measured 24 ​h after acquisition), and iv) enough evidence to conclude no task-specific stimulation-dependent differences in any of the neurophysiological measures (CSE and SICI).

The current acquisition and immediate retention findings (i.e., compelling evidence to suggest no generalised benefit of M1 anodal tDCS to motor acquisition and immediate retention) are in line with a number of studies reporting *no* significant beneficial effects (Chen et al., 2020; Lum et al., 2018; Mooney et al., 2019; Sobierajewicz et al., 2018). However, other studies reporting statistically significant beneficial effects of M1 anodal tDCS on explicit sequence acquisition (Cuypers et al., 2013; Saucedo Marquez et al., 2013; Stagg et al., 2011; Zimerman et al., 2013) and consolidation (Rumpf et al., 2017; Tecchio et al., 2010), point to the heterogeneity and equivocality of evidence. In the current study we only informed participants of the *presence* of an embedded sequence rather than the *composition* of that sequence. In contrast, the aforementioned studies reporting a beneficial effect of anodal tDCS utilized a sequence finger-tapping task as per Karni et al. (1995). There the sequence is displayed either on a computer screen, or participants are required to demonstrate explicit knowledge of the sequence *prior* to experimental manipulations. That is, the finger-tapping sequence had already been learned and possibly consolidated to some extent, prior to tDCS administration. Accordingly, it could be hypothesized that execution of an explicitly learnt finger-tapping task is underpinned primarily by motor control processes within the M1. Consequently, anodal tDCS applied over the M1 is likely to have resulted in beneficial effects for those studies utilizing such a finger-tapping task. In contrast, in the current study utilizing the SRTT, the sequence had not been learned prior to experimental manipulations in the acquisition phase, and the M1 may have played a less instrumental role in subserving task demands. Therefore, administering M1 anodal tDCS may have only affected a relatively small region of a broader neural network implicated in sequence learning. Indeed, recent research using tDCS has implicated the cerebellum (Ballard et al., 2019), prefrontal cortex (Greeley and Seidler, 2019; Lum et al., 2018), and posterior parietal cortex (Pollok et al., 2020) in sequence learning. Furthermore, the sequence finger-tapping task usually consists of a shorter 5-element sequence, whereas the SRTT we employed involved a longer 12-element sequence. To this end, recent research has implicated the M1 only in the learning of simple, and not complex, sequences (Clark et al., 2019), which conceivably affected the efficacy of M1 anodal tDCS in the current study. Taken together, these two aspects are likely to have greatly influenced the type of cognitive processing required by the older cohort in the present study during the explicit SRTT, and thereby possibly engaging a larger cortical network than that targeted using tDCS (i.e., primary motor cortex).

In younger adults, M1 anodal tDCS administered *before* SRTT acquisition has been reported to have a detrimental effect on explicit task performance (Amadi et al., 2015; Stagg et al., 2011). In the current study of older adults, anodal tDCS prior to motor skill acquisition, resulted in no particular detrimental or beneficial behavioural effect. This effect may be expected since no specific change in underlying neurophysiology (CSE or SICI) was observed in the current study after anodal tDCS compared to sham (Fig. 3, Fig. 4). Indeed, recent research has suggested no effect of a single session of M1 anodal tDCS (1 ​mA for 15 ​min) on CSE in healthy older adults (Ghasemian-Shirvan et al., 2020). Future studies may employ other tDCS stimulation parameters in healthy older adults (e.g., different intensity, duration, and/or a high-definition tDCS montage) to first establish meaningful neurophysiological changes prior to its utilization in behavioural settings.

Perhaps the most unexpected result was that anodal tDCS, compared to sham, applied during the acquisition phase lead to considerably *impaired* delayed retention, measured 24 ​h later, of the performance gains acquired during the SRTT training phase. That is, even though no stimulation specific (i.e., anodal vs. sham) differences were observed during motor skill acquisition, the detriments in retention suggest that early consolidation processes involved in explicit sequence learning were negatively affected by anodal tDCS. ‘Homeostatic plasticity’ is one plausible theory that may explain this detriment. Specifically, it has been shown that two excitability enhancing protocols, when administered in close temporal proximity, may lead to a *decrease* in cortical excitability due to homeostatic mechanisms that stabilize neural activity within a physiologically meaningful range (Karabanov et al., 2015). Given that both anodal tDCS and motor learning are thought to independently increase neuronal excitability, homeostatic mechanisms may have decreased excitability when applied concurrently leading to a degradation of underlying consolidation processes. Indeed, M1 anodal tDCS, applied immediately after an explicit sequence motor task, in healthy older adults also leads to significantly impaired consolidation (measured 6 ​h after acquisition) compared to sham stimulation (King et al., 2020). This and our study’s findings add credence to the notion of homeostatic plasticity mechanisms impairing retention in healthy older adults. Given that we report no differences in underlying neurophysiology (CSE or SICI) between anodal and sham conditions, this hypothesis may seem unlikely, however, another unmeasured neurophysiological mechanism (such as intracortical facilitation or interhemispheric inhibition) may have mediated this hypothesized homeostatic interaction.

From another perspective, these detrimental effects of anodal tDCS, applied concurrently with SRTT acquisition, on subsequent retention may also be partially explained by the notion of competition between implicit and explicit memory systems (Kantak et al., 2012). In the study by Kantak et al. (2012), anodal tDCS administered over the dorsal premotor cortex (PMd) during the acquisition phase of an *implicit* SRTT led to significantly less retention 24 ​h later compared to sham. The authors suggested that increased activation of the PMd – a region known to be involved in *explicit* learning – competed with offline stabilization mechanisms of the implicit memory system that support skill retention. Accordingly, it is conceivable that in the current study, activation of M1 – a region also associated in *implicit* learning (Pascual-Leone et al., 1994) – by means of anodal tDCS during an *explicit* SRTT, may have triggered competition between mechanisms involved in implicit and explicit memory systems that detrimentally affected retention. It is important to note, however, that off-line performance (*change* in performance over the 24 ​h period) remained relatively unaffected indicating a specific effect on early processes and not on long-term learning or stabilization mechanisms *per se*.

On a neurophysiological level, no *task-related* stimulation-dependent changes in CSE and SICI were observed for any of the three tDCS timing groups at any of the post-stimulation time-points. These results are in line with recent research on older adults (Mooney et al., 2019; DURING group in the current study) and is also consistent with research on younger adults reporting no statistically-significant change in MEP amplitude following concurrent anodal tDCS and motor acquisition (Amadi et al., 2015; Cabral et al., 2015) or when anodal tDCS is delivered after motor acquisition (Cabral et al., 2015; AFTER group in the current study). Moreover, another recent study (Ambrus et al., 2016) assessed anodal tDCS induced CSE changes *during* a SRTT in younger adults, as opposed to pre-post measurements, and reported no statistically-significant effects. For participants in the BEFORE group of the current study, though a generalised release of SICI was observed after anodal compared to sham stimulation – in line with research in older adults (Goodwill et al., 2013) – these effects were not modulated by motor skill acquisition or immediate retention. These null results contrast those of a study reporting *greater* levels of inhibition following a SRTT when preceded by anodal tDCS (akin to the BEFORE group in the current study) in a group of younger adults (Amadi et al., 2015). Considering the varied effects of anodal tDCS on SICI in younger and older adults (Heise et al., 2014), it seems likely that the aged status of the current cohort played a key role.

## 5 Future directions and conclusions

As alluded to earlier, tDCS stimulation to different cortical areas (cerebellum, prefrontal cortex, posterior parietal cortex) implicated in sequence learning may prove beneficial in providing further insights into how tDCS may facilitate behavioural learning. In addition, considering recent research suggesting the use of multi-session anodal tDCS (Dumel et al., 2016, 2018; Hashemirad et al., 2016; Yosephi et al., 2018), future studies may elucidate their utility on explicit motor acquisition and consolidation in older adults. Finally, given the known response variability of M1 anodal tDCS in older adults (Puri et al., 2016), future studies should consider taking into account various influential factors such as individual electric field characteristics (Albizu et al., 2020; Laakso et al., 2019), baseline GABA/glutamate ratios (Filmer et al., 2019), sensitivity to sham stimulation (Kortuem et al., 2019), expectation (Rabipour et al., 2018, 2019), attention (Yamaguchi et al., 2020), and genetic polymorphisms (Puri et al., 2015) to name a few.

In conclusion, the current study investigated - utilizing a robust randomized, double-blind, sham-controlled experiment - the efficacy and optimal timing of M1 anodal transcranial direct current stimulation in attenuating demonstrable behavioural deficits in healthy older adults. Probing this clinically relevant question our results suggest i) no generalised benefit of M1 anodal tDCS to motor acquisition and immediate retention, ii) not enough evidence for tDCS timing-based stimulation differences during motor acquisition and immediate retention, iii) a detrimental impact of anodal tDCS applied during motor acquisition on delayed retention, and iv) no task-specific stimulation-dependent differences in neurophysiological measures.

## Declaration of competing interest

The authors declare that they have no known competing financial interests or personal relationships that could have appeared to influence the work reported in this paper.
