# Supplementary material for: Timing-specific effects of single-session M1 anodal tDCS on motor sequence retention in healthy older adults
Source: Neuroimage Rep. 2021 Apr 12;1(1):100009. doi: 10.1016/j.ynirp.2021.100009 (PMC12172943; doi:10.1016/j.ynirp.2021.100009)
Supplement: Supplementary file 1 [file mmc1.docx]

**Supplementary Material 1**

**Methods**

*Participants*

Twenty-six adults (14 young adults; 12 older adults) were recruited from the university and local community. The Mini-Mental State Examination (Dick et al., 1984) was utilized to screen older participants for cognitive integrity and all participants scored within a normal range (score ≥ 26; Jacqmin-Gadda et al., 1997). Participants provided written informed consent prior to participation and all, except one older adult (left-hand dominant), declared right-hand dominance. The study was approved by the Tasmanian Human Research Ethics Committee Network and conducted in accordance with the Declaration of Helsinki.

*Serial reaction time task (SRTT)*

In the current experiment, two forms (implicit and explicit) of a SRTT were tested. The explicit version of the SRTT employed here was the same as that in the main experiment (see Section 2). For the implicit version (implicit SRTT), the task was presented as a reaction time task and, unlike the explicit SRTT, participants were *not* made aware of the presence of a repeated sequence and no change in stimulus colour (from black to blue) occurred for the first element of the repeating sequence.

In addition, sequence awareness tests were conducted to probe both a) free recall and b) recognition of the sequence. For free recall, participants pressed, on the keypad, as many consecutive elements of the sequence they believed had occurred during the task. Sequence recognition was tested using the fragment recognition test (Curran, 1997; Shanks and Johnstone, 1999) in which participants were asked how certain they were of having seen or not seen a 4-element fragment in the SRTT. Half of the 4-element fragments on the test were present in the SRTT, resulting in a total of twelve 4-element fragments based on the 12-element sequence (‘present’ fragments), with the other half of the tested 4-element fragments – constructed utilizing similar sequence constraints – not appearing in the SRTT (‘absent’ fragments). Participants responded, for each fragment, on a six-point scale ranging from certain (1), fairly certain (2), guessing (3) ‘seen’ to guessing (4), fairly certain (5), and certain (6) ‘not seen’.

*Experimental procedure*

Young (*n* =14) and older adults (*n* = 12) participated in two separate sessions of 1 h each, a minimum of 48 h apart, with both sessions conducted at the same time of day (± 30 min) for each participant. Considering the within-subjects design of this experiment, the implicit version of the SRTT was always administered in the first session and the explicit version in the second session. Participants completed 10 blocks for each version of the SRTT (non-invasive brain stimulation techniques were not administered in the current experiment), followed by sequence awareness tests to end each session (Supplementary Figure 1).

Supplementary Figure 1 – **Experimental procedure.** Twenty-six healthy adults (*n* = 14, young group; *n* = 12, older group) participated in two sessions, a minimum of 48 h apart, with session one always conducted using the *implicit* version of the SRTT and session two the *explicit* version. Both sessions consisted of 10 task blocks, lasting approximately 1 h each, ending with sequence awareness tests (free recall and fragment recognition).

*Data processing and analysis*

Reaction time data from both versions of the SRTT were subjected to the same processing techniques as those in the main experiment (see Section 2.6). For the free recall test, the number of consecutive elements that formed part of the correct sequence was quantified. In regards to the fragment recognition test, a difference between the average scores for ‘absent’ and ‘present’ fragments were computed (average ‘absent’ score – average ‘present’ score), with higher scores indicating greater sequence recognition.

Participants recalling greater than 5 elements on the implicit version of the SRTT (2 young adults) were excluded from further analyses. Sequence-specific learning was then subjected to a 3-way Bayesian ANOVA with factors of AGE GROUP (Young, Older), VERSION (Implicit, Explicit), and BLOCK (Blocks 1 to 10), probing age-related differences in performance between the two versions of the SRTT. Sequence awareness - number of elements recalled (free recall test) and sequence recognition scores (fragment recognition test) - were analysed using separate 2-way Bayesian ANOVAs with factors of AGE GROUP (Young, Older) and VERSION (Implicit, Explicit).

All parameters for the Bayesian ANOVAs were as described in the main article (see Section 2.7). Follow up Bayesian t-tests were conducted with the alternative hypothesis (subscript *1*) specified as the population means being different (i.e., ≠ 0) and the null hypothesis (subscript *0*) specified as the population means being equal (i.e., = 0). Thus, *BF_10_* indicates evidence for the alternative hypothesis compared to the null hypothesis and *BF_01_* indicates evidence for the null hypothesis compared to the alternative hypothesis. Both, “medium” (Cauchy distribution with scale = 0.707) and “ultrawide” (Cauchy distribution with scale = 1.414) prior specifications were used to check for Bayes factor robustness. Descriptive statistics are reported as means and 95% credible intervals (CIs: in square brackets), unless specified otherwise, and are used in all figures.

**Results**

Participants in the young (*n* = 12; mean age ± SD = 26.1 ± 4.8 years; 10 women and 2 men; all right-hand dominant) and older (*n* = 12; mean age ± SD = 66.3 ± 7.7 years; 8 women and 4 men; all, but one, right-hand dominant) cohorts completed both sessions without any issues and without visual evidence of any strategic slowing *within* blocks (see additional analyses titled ‘supp_1b_young’ and ‘supp_1b_older’ in the ‘supplementary_1’ folder on https://osf.io/pk8cv/). One young participant’s free recall data could not be collected due to technical difficulties.

*SRTT performance*

Here we concentrate on interaction effects involving AGE GROUP and task VERSION. The data provided very strong to extreme evidence for the exclusion of the 3-way interaction between AGE GROUP, VERSION, and BLOCK (*BF_med_excl_* = 40.92; *BF_uw_excl_* = 804.94) but provided extreme evidence for the *inclusion* of the 2-way interaction between AGE GROUP and VERSION (*BF_med_incl_* = 1.54e6; *BF_uw_incl_* = 8.99e5).

Follow-up Bayesian paired-samples t-tests revealed that, for young adults, the data provided extreme evidence (*BF_med_10_* = 772.67; *BF_uw_10_* = 1055.78) for sequence-specific learning being greater in the explicit (22.12%, [17.46 – 26.79]) compared to the implicit (9.72%, [5.75 – 13.69]) version, whereas for older adults, the data provided anecdotal evidence (*BF_med_10_* = 1.20; *BF_uw_01_* = 1.23) for a difference, or lack thereof, between explicit (14.51%, [9.01 – 20.1]) and implicit (9.84%, [7.76 – 11.92]) performance, as illustrated in Supplementary Figure 2 (top).

*Sequence awareness*

Free recall analyses suggested that the data provided moderate evidence for the inclusion of the AGE GROUP * VERSION interaction effect (*BF_med_incl_* = 6.29; *BF_uw_incl_* = 7.16). Follow-up Bayesian independent-samples t-tests revealed that, for implicit recall, there was anecdotal to moderate evidence (*BF_med_01_* = 2.28; *BF_uw_01_* = 3.80) for no difference between young (3.55 elements, [2.58 – 4.51]) and older (3.08 elements, [1.83 – 4.34]) participants, whereas the data provided strong to very strong evidence (*BF_med_10_* = 27.95; *BF_uw_10_* = 32.21) for young participants (10.42 elements, [8.92 – 11.91]) recalling more elements of the sequence than older participants (6.25 elements, [4.31 – 8.19]) after the explicit version of the task as depicted in Supplementary Figure 2 (bottom).

For sequence recognition, the data only provided anecdotal evidence for the main effect of AGE GROUP (*BF_med_excl_* = 1.09; *BF_uw_excl_* = 1.52) and the interaction effect of AGE GROUP * VERSION (*BF_med_incl_* = 1.16; *BF_uw_excl_* = 1.11).

Supplementary Figure 2 – **Behavioural outcomes.** For both older (no fill) and young (grey fill) age-groups, the ordinate depicts, on top, sequence-specific learning (difference in RT between random and sequence trials, as a proportion of RT on random trials, expressed as a %) and, on the bottom, number of consecutive elements correctly recalled as part of the sequence, for both implicit and explicit versions (abscissa). Error bars indicate 95% CIs around the mean.
