# Supplementary material for: Timing-specific effects of single-session M1 anodal tDCS on motor sequence retention in healthy older adults
Source: Neuroimage Rep. 2021 Apr 12;1(1):100009. doi: 10.1016/j.ynirp.2021.100009 (PMC12172943; doi:10.1016/j.ynirp.2021.100009)
Supplement: Supplementary file 2 [file mmc2.docx]

**Supplementary Material 2**

**Methods**

Similar to the methodology utilized in the experiment described in Supplementary Material 1, sequence awareness tests were conducted to probe both free recall and recognition of the sequence. Briefly, for free recall, participants pressed, on the keypad, as many consecutive elements of the sequence they believed had occurred during the task. Sequence recognition was tested using the fragment recognition test (Curran, 1997; Shanks and Johnstone, 1999) in which participants were asked how certain they were of having seen or not seen a 4-element fragment in the explicit SRTT. Half of the 4-element fragments on the test were present in the SRTT, resulting in a total of twelve 4-element fragments based on the 12-element sequence (‘present’ fragments), with the other half of the tested 4-element fragments – constructed utilizing similar sequence constraints – not appearing in the SRTT (‘absent’ fragments). Participants responded, for each fragment, on a six-point scale ranging from certain (1), fairly certain (2), guessing (3) ‘seen’ to guessing (4), fairly certain (5), and certain (6) ‘not seen’. Sequence awareness – free recall test and the fragment recognition test – was analysed using three-way Bayesian ANOVAs with factors of STIM (Anodal, Sham), SESSION (Session 1, Session 2), and GROUP (Before, During, After).

To investigate tDCS comfort, a tDCS sensations questionnaire (Fertonani et al., 2010) was administered at the end of each tDCS session. Participants rated 7 sensations (itching, pain, burning, warmth/heat, pinching, iron taste, and fatigue) on a 5-point scale (0 - ‘none’, 1 - ‘mild’, 2 - ‘moderate’, 3 - ‘considerable’, and 4 - ‘strong’). Data from the tDCS sensations questionnaire, collected after anodal and sham tDCS for all participants, was aggregated separately for each tDCS session ranging from 0 (score of 0 on all 7 sensations indicating absence of discomfort) to 28 (score of 4 on all 7 sensations indicating maximal discomfort) (Fertonani et al., 2015) and subjected to a two-way Bayesian ANOVA with factors of GROUP (Before, During, After) and STIM (Anodal, Sham).

All parameters for the Bayesian ANOVAs were as described in the main article (see Section 2.7). Descriptive statistics are reported as means and 95% credible intervals (CIs: in square brackets), unless specified otherwise.

**Results**

*Sequence awareness*

Three participant’s free recall data (two in the BEFORE and one in the DURING group) could not be collected due to technical difficulties.

No group- or stimulation-based differences in free recall (*BF_med_excl_* = 2.21 – 6.56; *BF_uw_excl_* = 3.91 – 19.82) or sequence recognition (*BF_med_excl_* = 1.17 – 8.64; *BF_uw_excl_* = 2.09 – 27.87) were observed as the data provided anecdotal to strong evidence for the exclusion of all main and interaction effects.

*tDCS discomfort*

No group-based differences in discomfort were observed (moderate evidence for the exclusion of GROUP main effect and GROUP * STIM interaction effect: *BF_med_excl_* = 3.64 – 3.69; *BF_uw_excl_* = 8.74 – 9.06). However, participants perceived slightly greater discomfort after anodal tDCS (2.02, [1.53 – 2.51]) than sham tDCS (1.29, [0.81 – 1.77]) as suggested by the strong evidence for the inclusion of the STIM main effect (*BF_med_incl_* = 20.02; *BF_uw_incl_* = 14.94).

Two-way ANOVAs, conducted on each sensation separately, revealed that the higher discomfort during anodal tDCS was primarily driven by higher ratings for ‘itchiness’ (strong evidence for inclusion of STIM main effect: *BF_med_incl_* = 83.84; *BF_uw_incl_* = 68.79) as reported in Supplementary Table 1. For all other sensations, evidence from the data ranged from providing anecdotal evidence for the inclusion of main and interactions effects (*BF_med_incl_* = 2.44; *BF_uw_incl_* = 1.58) to providing strong evidence for the exclusion of main and interaction effects (*BF_med_excl_* = 6.33; *BF_uw_excl_* = 18.03).

|  | Itchiness | Pain | Burning | Warmth  /Heat | Pinching | Iron Taste | Fatigue |
| --- | --- | --- | --- | --- | --- | --- | --- |
| Anodal | 0.65  [0.39 – 0.90] | 0.13  [0.03 – 0.22] | 0.35  [0.16 – 0.55] | 0.40  [0.24 – 0.55] | 0.13  [-0.004 – 0.25] | 0.02  [-0.02 – 0.06] | 0.35  [0.16 – 0.55] |
| Sham | 0.21  [0.06 – 0.36] | 0.13  [0.03 – 0.22] | 0.19  [0.05 – 0.33] | 0.48  [0.28 – 0.68] | 0.06  [-0.009 – 0.13] | 0.02  [-0.02 – 0.06] | 0.21  [0.06 – 0.36] |

Supplementary Table 1 – Mean and 95% CIs for each sensation in the tDCS sensations questionnaire collected after anodal and sham tDCS for all participants (*N* = 48).
